# Supplementary material for: Stiffness reduction and collagenase resistance of aging lungs measured using scanning acoustic microscopy
Source: PLoS One. 2022 Feb 17;17(2):e0263926. doi: 10.1371/journal.pone.0263926 (PMC8853515; doi:10.1371/journal.pone.0263926)
Supplement: S1 Table — (DOCX) [file pone.0263926.s001.docx]

**S1 Table. One-way ANOVA for SOS values with different fixation methods**

| a.Bronchiole |  |  |  |  |  |  |  |  |
| --- | --- | --- | --- | --- | --- | --- | --- | --- |
| Tissue samples | n | mean m/s | SD | SE |  |  |  |  |
| Fresh | 25 | 1625.7 | 36.88 | 7.38 |  |  |  |  |
| Formalin | 25 | 1638.2 | 27.04 | 5.41 |  |  |  |  |
| FFPE | 25 | 1685.7 | 25.25 | 5.05 |  |  |  |  |
|  |  |  |  |  |  |  |  |  |
| Test for homogeneity of variance |  |  |  |  |  |  |  |  |
| Bartlett's test |  |  | Levine's test |  |  |  |  |  |
| chi-square | df | P-value | F-value | freedom1 | freedom2 | P-value |  |  |
| 4.04 | 2 | 0.13 | 3.06 | 2 | 72 | 0.05 |  |  |
|  |  |  |  |  |  |  |  |  |
| Analysis of variance |  |  |  |  |  |  |  |  |
| Source of variation | Sum of squares | df | mean square | F-value | P-value |  |  |  |
| Between groups | 50236.2 | 2 | 25118.1 | 27.6177 | P < 0.001 | ** |  |  |
| Within groups | 65483.5 | 72 | 909.5 |  |  |  |  |  |
| Total | 115719.7 | 74 |  |  |  |  |  |  |
|  |  |  |  |  |  |  |  |  |
| Multiple comparison test (Tukey) |  |  |  |  |  |  |  |  |
| Group A | Group B | Mean A | Mean B | Difference | SE | Statistics | P-value |  |
| Fresh-Bro | Form-Bro | 1625.7 | 1638.2 | 12.6 | 8.53 | 1.4720 | 0.3103 |  |
| Fresh-Bro | FFPE-Bro | 1625.7 | 1685.7 | 60.1 | 8.53 | 7.0449 | P < 0.001 | ** |
| Form-Bro | FFPE-Bro | 1638.2 | 1685.7 | 47.5 | 8.53 | 5.5728 | P < 0.001 | ** |
|  |  |  |  |  |  |  |  |  |
| b.Arteriole |  |  |  |  |  |  |  |  |
| Tissue samples | n | mean m/s | SD | SE |  |  |  |  |
| Fresh | 25 | 1642.7 | 33.36 | 6.67 |  |  |  |  |
| Formalin | 25 | 1668.6 | 45.85 | 9.17 |  |  |  |  |
| FFPE | 25 | 1713.2 | 31.83 | 6.37 |  |  |  |  |
|  |  |  |  |  |  |  |  |  |
| Test for homogeneity of variance |  |  |  |  |  |  |  |  |
| Bartlett's test |  |  | Levine's test |  |  |  |  |  |
| chi-square | df | P-value | F-value | freedom1 | freedom2 | P-value |  |  |
| 3.93 | 2 | 0.14 | 1.63 | 2 | 72 | 0.20 |  |  |
|  |  |  |  |  |  |  |  |  |
| Analysis of variance |  |  |  |  |  |  |  |  |
| Source of variation | Sum of squares | df | mean square | F-value | P-value |  |  |  |
| Between groups | 63651.7 | 2 | 31825.8 | 22.6 | P < 0.001 | ** |  |  |
| Within groups | 101483.9 | 72 | 1409.5 |  |  |  |  |  |
| Total | 165135.6 | 74 |  |  |  |  |  |  |
|  |  |  |  |  |  |  |  |  |
| Multiple comparison test (Tukey) |  |  |  |  |  |  |  |  |
| Group A | Group B | Mean A | Mean B | Difference | SE | Statistics | P-value |  |
| Fresh-Art | Form-Art | 1642.7 | 1668.6 | 26.0 | 10.6 | 2.4 | 0.0442 | * |
| Fresh-Art | FFPE-Art | 1642.7 | 1713.2 | 70.5 | 10.6 | 6.6 | P < 0.001 | ** |
| Form-Art | FFPE-Art | 1668.6 | 1713.2 | 44.6 | 10.6 | 4.2 | P < 0.001 | ** |
|  |  |  |  |  |  |  | |  |
| c.Alveoli |  |  |  |  |  |  |  |  |
| Tissue samples | n | mean m/s | SD | SE |  |  |  |  |
| Fresh | 25 | 1542.34 | 23.89 | 4.78 |  |  |  |  |
| Formalin | 25 | 1583.60 | 30.16 | 6.03 |  |  |  |  |
| FFPE | 25 | 1629.93 | 41.87 | 8.55 |  |  |  |  |
|  |  |  |  |  |  |  |  |  |
| Test for homogeneity of variance |  |  |  |  |  |  |  |  |
| Bartlett's test |  |  | Levine's test |  |  |  |  |  |
| chi-square | df | P-value | F-value | freedom1 | freedom2 | P-value |  |  |
| 7.37 | 2 | 0.03 | 3.75 | 2 | 72 | 0.03 |  |  |
|  |  |  |  |  |  |  |  |  |
| Analysis of variance |  |  |  |  |  |  |  |  |
| Source of variation | Sum of squares | df | mean square | F-value | P-value |  |  |  |
| Between groups | 93985.8 | 2 | 46992.9 | 44.0 | P < 0.001 | ** |  |  |
| Within groups | 75840.4 | 72 | 1068.2 |  |  |  |  |  |
| Total | 169826.2 | 74 |  |  |  |  |  |  |
|  |  |  |  |  |  |  |  |  |
| Multiple comparison test (Tukey) |  |  |  |  |  |  |  |  |
| Group A | Group B | Mean A | Mean B | Difference | SE | Statistics | P-value |  |
| Fresh-Alv | Form-Alv | 1542.3 | 1583.6 | 41.3 | 9.24 | 4.46 | P < 0.001 | ** |
| Fresh-Alv | FFPE-Alv | 1542.3 | 1629.9 | 87.6 | 9.34 | 9.38 | P < 0.001 | ** |
| Form-Alv | FFPE-Alv | 1583.6 | 1629.9 | 46.3 | 9.34 | 4.96 | P < 0.001 | ** |

Form; formalin, FFPE; formalin-fixed, paraffin-embedded, df, degrees of freedom, *：P<0.05 **：P<0.01
