# Supplementary material for: Stiffness reduction and collagenase resistance of aging lungs measured using scanning acoustic microscopy
Source: PLoS One. 2022 Feb 17;17(2):e0263926. doi: 10.1371/journal.pone.0263926 (PMC8853515; doi:10.1371/journal.pone.0263926)
Supplement: S2 Table — (DOCX) [file pone.0263926.s002.docx]

**S2 Table. One-way ANOVA for SOS values in different age groups**

| Bronchiole | Test for homogeneity of variance | |  |  |  |  |  |  |  |  |
| --- | --- | --- | --- | --- | --- | --- | --- | --- | --- | --- |
|  | Bartlett’s test |  |  | Levine's test |  |  |  |  |  |  |
|  | chi-square | df | P-value | F-value | freedom1 | freedum2 | P-value |  |  |  |
|  | 0.7550 | 2 | 0.69 | 0.37 | 2 | 145 | 0.69 |  |  |  |
|  |  |  |  |  |  |  |  |  |  |  |
|  | Analysis of variance | |  |  |  |  |  |  |  |  |
|  | Source of variation | Sum of squares | df | mean square | F-value | P-value | **：P<0.01 |  |  |  |
|  | Between group | 95285.4 | 2 | 47642.7 | 20.50 | P < 0.001 | ** |  |  |  |
|  | Within groups | 337003.4 | 145 | 2324.2 |  |  |  |  |  |  |
|  | Total | 432288.8 | 147 |  |  |  |  |  |  |  |
|  |  |  |  |  |  |  |  |  |  |  |
|  | Multiple comparison test | |  |  |  |  |  |  |  |  |
|  | Method | Group A | Group B | Mean A | Mean B | Difference | SE | Statistics | P-value |  |
|  | Tukey-Kramer | Y‐Bronchiole | M‐Bronchiole | 1695.5 | 1641.4 | 54.2 | 9.69 | 5.59 | P < 0.001 | ** |
|  |  | Y‐Bronchiole | O‐Bronchiole | 1695.5 | 1641.9 | 53.6 | 9.74 | 5.51 | P < 0.001 | ** |
|  |  | M‐Bronchiole | O‐Bronchiole | 1641.4 | 1641.9 | 0.5 | 9.69 | 0.06 | 0.9983 |  |
|  |  |  |  |  |  |  |  |  |  |  |
| Arteriole | Test for homogeneity of variance | | |  |  |  |  |  |  |  |
|  | Bartlett’s test |  |  | Levine's test |  |  |  |  |  |  |
|  | chi-square | df | P-value | F-value | freedom1 | freedum2 | P-value |  |  |  |
|  | 3.0684 | 2 | 0.22 | 2.62 | 2 | 147 | 0.0764 |  |  |  |
|  |  |  |  |  |  |  |  |  |  |  |
|  | Analysis of variance | |  |  |  |  |  |  |  |  |
|  | Source of variation | Sum of squares | df | mean square | F-value | P-value | **：P<0.01 |  |  |  |
|  | Between group | 111566.8 | 2 | 55783.4 | 14.51 | P < 0.001 | ** |  |  |  |
|  | Within groups | 565187.6 | 147 | 3844.8 |  |  |  |  |  |  |
|  | Total | 676754.5 | 149 |  |  |  |  |  |  |  |
|  |  |  |  |  |  |  |  |  |  |  |
|  | Multiple comparison test | |  |  |  |  |  |  |  |  |
|  | Method | Group A | Group B | Mean A | Mean B | Difference | SE | Statistics | P-value |  |
|  | Tukey-Kramer | Y-Arterioles | M-Arterioles | 1753.9 | 1692.5 | 61.4 | 12.40 | 4.95 | P < 0.001 | ** |
|  |  | Y-Arterioles | O-Arterioles | 1753.9 | 1700.4 | 53.5 | 12.40 | 4.32 | P < 0.001 | ** |
|  |  | M-Arterioles | O-Arterioles | 1692.5 | 1700.4 | 7.8 | 12.40 | 0.63 | 0.8037 |  |
|  |  |  |  |  |  |  |  |  |  |  |
| Alveoli | Test for homogeneity of variance | | |  |  |  |  |  |  |  |
|  | Bartlett’s test |  |  | Levine's test |  |  |  |  |  |  |
|  | chi-square | df | P-value | F-value | freedom1 | freedum2 | P-value |  |  |  |
|  | 6.8756 | 2 | 0.032 | 2.48 | 2 | 144 | 0.087 |  |  |  |
|  |  |  |  |  |  |  |  |  |  |  |
|  | Analysis of variance | |  |  |  |  |  |  |  |  |
|  | Source of variation | Sum of squares | df | mean square | F-value | P-value | **：P<0.01 |  |  |  |
|  | Between group | 41697.9 | 2 | 20848.9 | 12.72 | P < 0.001 | ** |  |  |  |
|  | Within groups | 235948.1 | 144 | 1638.5 |  |  |  |  |  |  |
|  | Total | 277646.0 | 146 |  |  |  |  |  |  |  |
|  |  |  |  |  |  |  |  |  |  |  |
|  | Multiple comparison test | |  |  |  |  |  |  |  |  |
|  | Method | Group A | Group B | Mean A | Mean B | Difference | SE | Statistics | P-value |  |
|  | Tukey-Kramer | Y-Alveoli | M-Alveoli | 1646.9 | 1620.0 | 26.92 | 8.18 | 3.29 | 0.0036 | ** |
|  |  | Y-Alveoli | O-Alveoli | 1646.9 | 1606.1 | 40.81 | 8.22 | 4.96 | P < 0.001 | ** |
|  |  | M-Alveoli | O-Alveoli | 1620.0 | 1606.1 | 13.89 | 8.14 | 1.71 | 0.2062 |  |
