# Supplementary material for: Stiffness reduction and collagenase resistance of aging lungs measured using scanning acoustic microscopy
Source: PLoS One. 2022 Feb 17;17(2):e0263926. doi: 10.1371/journal.pone.0263926 (PMC8853515; doi:10.1371/journal.pone.0263926)
Supplement: S3 Table — (DOCX) [file pone.0263926.s003.docx]

**S3 Table. One-way ANOVA for collagenase effects on SOS values in different age groups and different durations**

| Arteriole | Y-0h | Y-1.5h | Y-3h | M-0h | M-1.5h | M-3h | O-0h | O-1.5h | O-3h |
| --- | --- | --- | --- | --- | --- | --- | --- | --- | --- |
| n | 50 | 50 | 49 | 50 | 50 | 50 | 50 | 48 | 50 |
| mean | 1753.9 | 1694.3 | 1669.0 | 1692.5 | 1660.3 | 1660.2 | 1700.4 | 1669.3 | 1663.7 |
| SD | 57.5 | 43.9 | 37.4 | 56.8 | 49.3 | 50.8 | 70.7 | 72.3 | 67.4 |
| SE | 8.13 | 6.21 | 5.35 | 8.03 | 6.97 | 7.18 | 10.00 | 10.44 | 9.54 |
|  |  |  |  |  |  |  |  |  |  |
| Bronchiole | Y-0h | Y-1.5h | Y-3h | M-0h | M-1.5h | M-3h | O-0h | O-1.5h | O-3h |
| n | 49 | 49 | 50 | 50 | 50 | 50 | 49 | 50 | 50 |
| mean | 1695.5 | 1665.6 | 1648.9 | 1641.4 | 1613.8 | 1615.1 | 1641.9 | 1631.9 | 1617.1 |
| SD | 50.8 | 39.9 | 46.6 | 44.9 | 48.1 | 43.1 | 48.9 | 57.0 | 66.2 |
| SE | 7.3 | 5.7 | 6.6 | 6.3 | 6.8 | 6.1 | 7.0 | 8.1 | 9.4 |
|  |  |  |  |  |  |  |  |  |  |
| Alveoli | Y-0h | Y-1.5h | Y-3h | M-0h | M-1.5h | M-3h | O-0h | O-1.5h | O-3h |
| n | 48 | 50 | 50 | 50 | 50 | 49 | 49 | 50 | 50 |
| mean | 1646.9 | 1623.3 | 1610.0 | 1620.0 | 1587.5 | 1590.7 | 1606.1 | 1599.6 | 1595.9 |
| SD | 32.1 | 36.9 | 33.8 | 47.2 | 37.8 | 31.0 | 40.5 | 43.6 | 42.4 |
| SE | 4.6 | 5.2 | 4.8 | 6.7 | 5.3 | 4.4 | 5.8 | 6.2 | 6.0 |
| ARTERIOLES |  |  |  |  |  |  |  |  |  |
| Y-arteriole |  |  |  |  |  |  |  |  |  |
| Test for homogeneity of variance | |  |  |  |  |  |  |  |  |
| Bartlett’s test |  |  | Levine's test |  |  |  |  |  |  |
| chi-square | df | P-value | F-value | freedom1 | freedom2 | P-value |  |  |  |
| 9.2189 | 2 | 0.0100 | 5.89 | 2 | 146 | 0.0035 |  |  |  |
|  |  |  |  |  |  |  |  |  |  |
| Analysis of variance |  |  |  |  |  |  |  |  |  |
| Source of variation | Sum of squares | df | mean square | F-value | P-value | **：P<0.01 |  |  |  |
| Between group | 188727.9 | 2 | 94363.9 | 42.55 | P < 0.001 | ** |  |  |  |
| Within groups | 323774.0 | 146 | 2217.63 |  |  |  |  |  |  |
| Total | 512501.9 | 148 |  |  |  |  |  |  |  |
|  |  |  |  |  |  |  |  |  |  |
| Multiple comparison test | |  |  |  |  |  |  |  |  |
| Method | Group A | Group B | Mean A | Mean B | Difference | SE | Statistics | P-value |  |
| Tukey-Kramer | Y-0h | Y-1.5h | 1753.9 | 1694.3 | 59.6 | 9.4 | 6.33 | P < 0.001 | ** |
|  | Y-0h | Y-3h | 1753.9 | 1669.0 | 84.9 | 9.5 | 8.97 | P < 0.001 | ** |
|  | Y-1.5h | Y-3h | 1694.3 | 1669.0 | 25.3 | 9.5 | 2.67 | 0.0226 | * |
|  |  |  |  |  |  |  |  |  |  |
|  | F-value | freedom1 | freedum2 | P-value | *：P<0.05 **：P<0.01 | |  |  |  |
| Welch | 37.8 | 2 | 95.1290 | P < 0.001 | ** |  |  |  |  |
| Brown-Forsythe | 42.7 | 2 | 129.8024 | P < 0.001 | ** |  |  |  |  |
|  |  |  |  |  |  |  |  |  |  |
| M-arteriole |  |  |  |  |  |  |  |  |  |
| Test for homogeneity of variance | |  |  |  |  |  |  |  |  |
| Bartlett’s test |  |  | Levine's test |  |  |  |  |  |  |
| chi-square | df | P-value | F-value | freedom1 | freedom2 | P-value |  |  |  |
| 1.1175 | 2 | 0.57 | 0.90 | 2 | 147 | 0.4105 |  |  |  |
|  |  |  |  |  |  |  |  |  |  |
| Analysis of variance |  |  |  |  |  |  |  |  |  |
| Source of variation | Sum of squares | df | mean square | F-value | P-value | **：P<0.01 |  |  |  |
| Between group | 34790.3 | 2 | 17395.2 | 6.34 | 0.0023 | ** |  |  |  |
| Within groups | 403342.4 | 147 | 2743.8 |  |  |  |  |  |  |
| Total | 438132.7 | 149 |  |  |  |  |  |  |  |
|  |  |  |  |  |  |  |  |  |  |
| Multiple comparison test | |  |  |  |  |  |  |  |  |
| Method | Group A | Group B | Mean A | Mean B | Difference | SE | Statistics | P-value |  |
| Tukey-Kramer | M-0h | M-1.5h | 1692.5 | 1660.3 | 32.2 | 10.48 | 3.08 | 0.0070 | ** |
|  | M-0h | M-3h | 1692.5 | 1660.2 | 32.4 | 10.48 | 3.09 | 0.0067 | ** |
|  | M-1.5h | M-3h | 1660.3 | 1660.2 | 0.1 | 10.48 | 0.01 | 0.9999 |  |
|  |  |  |  |  |  |  |  |  |  |
|  | F-value | freedom1 | freedom2 | P-value | *：P<0.05 **：P<0.01 | |  |  |  |
| Welch | 5.79 | 2 | 97.6612 | 0.0042 | ** |  |  |  |  |
| Brown-Forsythe | 6.34 | 2 | 144.6869 | 0.0023 | ** |  |  |  |  |
|  |  |  |  |  |  |  |  |  |  |
| O-arterioles |  |  |  |  |  |  |  |  |  |
| Test for homogeneity of variance | |  |  |  |  |  |  |  |  |
| Bartlett’s test |  |  | Levine's test |  |  |  |  |  |  |
| chi-square | df | P-value | F-value | freedom1 | freedom2 | P-value |  |  |  |
| 0.24 | 2 | 0.89 | 0.057 | 2 | 145 | 0.9445 |  |  |  |
|  |  |  |  |  |  |  |  |  |  |
| Analysis of variance |  |  |  |  |  |  |  |  |  |
| Source of variation | Sum of squares | df | mean square | F-value | P-value | **：P<0.01 |  |  |  |
| Between group | 38909.6 | 2 | 19454.8 | 3.95 | 0.021 | * |  |  |  |
| Within groups | 713759.3 | 145 | 4922.5 |  |  |  |  |  |  |
| Total | 752668.9 | 147 |  |  |  |  |  |  |  |
|  |  |  |  |  |  |  |  |  |  |
| Multiple comparison test | |  |  |  |  |  |  |  |  |
| Method | Group A | Group B | Mean A | Mean B | Difference | SE | Statistics | P-value |  |
| Tukey-Kramer | O-0h | O-1.5h | 1700.4 | 1669.3 | 31.1 | 14.2 | 2.19 | 0.076 |  |
|  | O-0h | O-3h | 1700.4 | 1663.7 | 36.7 | 14.0 | 2.61 | 0.027 | * |
|  | O-1.5h | O-3h | 1669.3 | 1663.7 | 5.6 | 14.2 | 0.39 | 0.918 |  |
|  |  |  |  |  |  |  |  |  |  |
|  | F-value | freedom1 | freedom2 | P-value | *：P<0.05 **：P<0.01 | |  |  |  |
| Welch | 3.95 | 2 | 96.4 | 0.023 | * |  |  |  |  |
| Brown-Forsythe | 3.95 | 2 | 144.0 | 0.021 | * |  |  |  |  |
|  |  |  |  |  |  |  |  |  |  |
| BRONCHIOLES |  |  |  |  |  |  |  |  |  |
| Y-bronchiole |  |  |  |  |  |  |  |  |  |
| Test for homogeneity of variance | |  |  |  |  |  |  |  |  |
| Bartlett’s test |  |  | Levine's test |  |  |  |  |  |  |
| chi-square | df | P-value | F-value | freedom1 | freedom2 | P-value |  |  |  |
| 2.77 | 2 | 0.25 | 1.24 | 2 | 145 | 0.29 |  |  |  |
|  |  |  |  |  |  |  |  |  |  |
| Analysis of variance |  |  |  |  |  |  |  |  |  |
| Source of variation | Sum of squares | df | mean square | F-value | P-value | **：P<0.01 |  |  |  |
| Between group | 55205.7 | 2 | 27602.8 | 13.07 | P < 0.001 | ** |  |  |  |
| Within groups | 306194.2 | 145 | 2111.7 |  |  |  |  |  |  |
| Total | 361399.9 | 147 |  |  |  |  |  |  |  |
|  |  |  |  |  |  |  |  |  |  |
| Multiple comparison test | |  |  |  |  |  |  |  |  |
| Method | Group A | Group B | Mean A | Mean B | Difference | SE | Statistics | P-value |  |
| Tukey-Kramer | Y-0h | Y-1.5h | 1695.5 | 1665.6 | 29.9 | 9.28 | 3.22 | 0.0044 | ** |
|  | Y-0h | Y-3h | 1695.5 | 1648.9 | 46.7 | 9.24 | 5.05 | P < 0.001 | ** |
|  | Y-1.5h | Y-3h | 1665.6 | 1648.9 | 16.7 | 9.24 | 1.81 | 0.1692 |  |
|  |  |  |  |  |  |  |  |  |  |
|  | F-value | freedom1 | freedom2 | P-value | *：P<0.05 **：P<0.01 | |  |  |  |
| Welch | 11.4 | 2 | 95.7 | P < 0.001 | ** |  |  |  |  |
| Brown-Forsythe | 13.1 | 2 | 139.8 | P < 0.001 | ** |  |  |  |  |
|  |  |  |  |  |  |  |  |  |  |
| M-bronchiole |  |  |  |  |  |  |  |  |  |
| Test for homogeneity of variance | |  |  |  |  |  |  |  |  |
| Bartlett’s test |  |  | Levine's test |  |  |  |  |  |  |
| chi-square | df | P-value | F-value | freedom1 | freedom2 | P-value |  |  |  |
| 0.60 | 2 | 0.74 | 0.15 | 2 | 147 | 0.86 |  |  |  |
|  |  |  |  |  |  |  |  |  |  |
| Analysis of variance |  |  |  |  |  |  |  |  |  |
| Source of variation | Sum of squares | df | mean square | F-value | P-value | **：P<0.01 |  |  |  |
| Between group | 24098.1 | 2 | 12049.0 | 5.85 | 0.0036 | ** |  |  |  |
| Within groups | 302736.3 | 147 | 2059.4 |  |  |  |  |  |  |
| Total | 326834.4 | 149 |  |  |  |  |  |  |  |
|  |  |  |  |  |  |  |  |  |  |
| Multiple comparison test | |  |  |  |  |  |  |  |  |
| Method | Group A | Group B | Mean A | Mean B | Difference | SE | Statistics | P-value |  |
| Tukey-Kramer | M-0h | M-1.5h | 1641.4 | 1613.8 | 27.5 | 9.08 | 3.03 | 0.0081 | ** |
|  | M-0h | M-3h | 1641.4 | 1615.1 | 26.2 | 9.08 | 2.89 | 0.0123 | * |
|  | M-1.5h | M-3h | 1613.8 | 1615.1 | 1.3 | 9.08 | 0.14 | 0.9889 |  |
|  |  |  |  |  |  |  |  |  |  |
|  | F-value | freedom1 | freedom2 | P-value | *：P<0.05 **：P<0.01 | |  |  |  |
| Welch | 5.87 | 2 | 97.8 | 0.0039 | ** |  |  |  |  |
| Brown-Forsythe | 5.85 | 2 | 145.8 | 0.0036 | ** |  |  |  |  |
|  |  |  |  |  |  |  |  |  |  |
| O-bronchiole |  |  |  |  |  |  |  |  |  |
| Test for homogeneity of variance | |  |  |  |  |  |  |  |  |
| Bartlett’s test |  |  | Levine's test |  |  |  |  |  |  |
| chi-square | df | P-value | F-value | freedom1 | freedom2 | P-value |  |  |  |
| 4.3926 | 2 | 0.1112 | 0.8677 | 2 | 146 | 0.4221 |  |  |  |
|  |  |  |  |  |  |  |  |  |  |
| Analysis of variance |  |  |  |  |  |  |  |  |  |
| Source of variation | Sum of squares | df | mean square | F-value | P-value | **：P<0.01 |  |  |  |
| Between group | 15487.2 | 2 | 7743.6 | 2.31 | 0.103 |  |  |  |  |
| Within groups | 489001.1 | 146 | 3349.3 |  |  |  |  |  |  |
| Total | 504488.3 | 148 |  |  |  |  |  |  |  |
|  |  |  |  |  |  |  |  |  |  |
| Multiple comparison test | |  |  |  |  |  |  |  |  |
| Method | Group A | Group B | Mean A | Mean B | Difference | SE | Statistics | P-value |  |
| Tukey-Kramer | O-0h | O-1.5h | 1641.9 | 1631.9 | 10.0 | 11.63 | 0.86 | 0.6692 |  |
|  | O-0h | O-3h | 1641.9 | 1617.1 | 24.8 | 11.63 | 2.13 | 0.0864 |  |
|  | O-1.5h | O-3h | 1631.9 | 1617.1 | 14.9 | 11.57 | 1.29 | 0.4054 |  |
|  |  |  |  |  |  |  |  |  |  |
|  | F-value | freedom1 | freedom2 | P-value | *：P<0.05 **：P<0.01 | |  |  |  |
| Welch | 2.25 | 2 | 96.2 | 0.111 |  |  |  |  |  |
| Brown-Forsythe | 2.32 | 2 | 138.4 | 0.102 |  |  |  |  |  |
|  |  |  |  |  |  |  |  |  |  |
| ALVEOLI |  |  |  |  |  |  |  |  |  |
| Y-alveoli |  |  |  |  |  |  |  |  |  |
| Test for homogeneity of variance | |  |  |  |  |  |  |  |  |
| Bartlett’s test |  |  | Levine's test |  |  |  |  |  |  |
| chi-square | df | P-value | F-value | freedom1 | freedom2 | P-value |  |  |  |
| 0.99 | 2 | 0.61 | 0.65 | 2 | 145 | 0.52 |  |  |  |
|  |  |  |  |  |  |  |  |  |  |
| Analysis of variance |  |  |  |  |  |  |  |  |  |
| Source of variation | Sum of squares | df | mean square | F-value | P-value | **：P<0.01 |  |  |  |
| Between group | 34120.5 | 2 | 17060.2 | 14.5 | P < 0.001 | ** |  |  |  |
| Within groups | 171057.9 | 145 | 1179.7 |  |  |  |  |  |  |
| Total | 205178.3 | 147 |  |  |  |  |  |  |  |
|  |  |  |  |  |  |  |  |  |  |
| Multiple comparison test | |  |  |  |  |  |  |  |  |
| Method | Group A | Group B | Mean A | Mean B | Difference | SE | Statistics | P-value |  |
| Tukey-Kramer | Y-0h | Y-1.5h | 1646.9 | 1623.3 | 23.6 | 6.94 | 3.39 | 0.0026 | ** |
|  | Y-0h | Y-3h | 1646.9 | 1610.0 | 36.9 | 6.94 | 5.32 | P < 0.001 | ** |
|  | Y-1.5h | Y-3h | 1623.3 | 1610.0 | 13.4 | 6.87 | 1.95 | 0.1295 |  |
|  |  |  |  |  |  |  |  |  |  |
|  | F-value | freedom1 | freedom2 | P-value | *：P<0.05 **：P<0.01 | |  |  |  |
| Welch | 15.7 | 2 | 96.5 | P < 0.001 | ** |  |  |  |  |
| Brown-Forsythe | 14.5 | 2 | 143.6 | P < 0.001 | ** |  |  |  |  |
|  |  |  |  |  |  |  |  |  |  |
| M-alveoli |  |  |  |  |  |  |  |  |  |
| Test for homogeneity of variance | |  |  |  |  |  |  |  |  |
| Bartlett’s test |  |  | Levine's test |  |  |  |  |  |  |
| chi-square | df | P-value | F-value | freedom1 | freedom2 | P-value |  |  |  |
| 8.42 | 2 | 0.015 | 3.16 | 2 | 146 | 0.045 |  |  |  |
|  |  |  |  |  |  |  |  |  |  |
| Analysis of variance |  |  |  |  |  |  |  |  |  |
| Source of variation | Sum of squares | df | mean square | F-value | P-value | **：P<0.01 |  |  |  |
| Between group | 31956.6 | 2 | 15978.3 | 10.37 | P < 0.001 | ** |  |  |  |
| Within groups | 225021.9 | 146 | 1541.2 |  |  |  |  |  |  |
| Total | 256978.5 | 148 |  |  |  |  |  |  |  |
|  |  |  |  |  |  |  |  |  |  |
| Multiple comparison test | |  |  |  |  |  |  |  |  |
| Method | Group A | Group B | Mean A | Mean B | Difference | SE | Statistics | P-value |  |
| Tukey-Kramer | 0h | 1.5h | 1620.0 | 1587.5 | 32.5 | 7.85 | 4.13 | P < 0.001 | ** |
|  | 0h | 3h | 1620.0 | 1590.7 | 29.3 | 7.89 | 3.71 | P < 0.001 | ** |
|  | 1.5h | 3h | 1587.5 | 1590.7 | 3.2 | 7.89 | 0.40 | 0.91 |  |
|  |  |  |  |  |  |  |  |  |  |
|  | F-value | freedom1 | freedom2 | P-value | *：P<0.05 **：P<0.01 | |  |  |  |
| Welch | 8.38 | 2 | 95.1 | P < 0.001 | ** |  |  |  |  |
| Brown-Forsythe | 10.41 | 2 | 131.7 | P < 0.001 | ** |  |  |  |  |
|  |  |  |  |  |  |  |  |  |  |
| O-alveoli |  |  |  |  |  |  |  |  |  |
| Test for homogeneity of variance | |  |  |  |  |  |  |  |  |
| Bartlett’s test |  |  | Levine's test |  |  |  |  |  |  |
| chi-square | df | P-value | F-value | freedom1 | freedom2 | P-value |  |  |  |
| 0.28 | 2 | 0.87 | 0.066 | 2 | 146 | 0.94 |  |  |  |
|  |  |  |  |  |  |  |  |  |  |
| Analysis of variance |  |  |  |  |  |  |  |  |  |
| Source of variation | Sum of squares | df | mean square | F-value | P-value | **：P<0.01 |  |  |  |
| Between group | 2608.5 | 2 | 1304.3 | 0.73 | 0.48 |  |  |  |  |
| Within groups | 260259.0 | 146 | 1782.6 |  |  |  |  |  |  |
| Total | 262867.5 | 148 |  |  |  |  |  |  |  |
|  |  |  |  |  |  |  |  |  |  |
| Multiple comparison test | |  |  |  |  |  |  |  |  |
| Method | Group A | Group B | Mean A | Mean B | Difference | SE | Statistics | P-value |  |
| Tukey-Kramer | 0h | 1.5h | 1606.1 | 1599.6 | 6.51 | 8.49 | 0.77 | 0.72 |  |
|  | 0h | 3h | 1606.1 | 1595.9 | 10.14 | 8.49 | 1.19 | 0.46 |  |
|  | 1.5h | 3h | 1599.6 | 1595.9 | 3.64 | 8.44 | 0.43 | 0.90 |  |
|  |  |  |  |  |  |  |  |  |  |
|  | F-value | freedom1 | freedom2 | P-value |  |  |  |  |  |
| Welch | 0.76 | 2 | 97.3 | 0.47 |  |  |  |  |  |
| Brown-Forsythe | 0.73 | 2 | 145.7 | 0.48 |  |  |  |  |  |
